# *CCDC78*: Unveiling the Function of a Novel Gene Associated with Hereditary Myopathy

**DOI:** 10.3390/cells13171504

**Published:** 2024-09-08

**Authors:** Diego Lopergolo, Gian Nicola Gallus, Giuseppe Pieraccini, Francesca Boscaro, Gianna Berti, Giovanni Serni, Nila Volpi, Patrizia Formichi, Silvia Bianchi, Denise Cassandrini, Vincenzo Sorrentino, Daniela Rossi, Filippo Maria Santorelli, Nicola De Stefano, Alessandro Malandrini

**Affiliations:** 1 Department of Medicine, Surgery and Neurosciences, University of Siena, 53100 Siena, Italy; 2 UOC Neurologia, Azienda Ospedaliero-Universitaria Senese, 53100 Siena, Italy; 3 CISM—Mass Spectrometry Centre, University of Florence, 50139 Florence, Italy; 4 Molecular Medicine for Neurodegenerative and Neuromuscular Diseases Unit, IRCCS Stella Maris Foundation, 56128 Pisa, Italy; 5 Department of Molecular and Developmental Medicine, University of Siena, 53100 Siena, Italy; 6 Interdepartmental Program of Molecular Diagnosis and Pathogenetic Mechanisms of Rare Genetic Diseases, Azienda Ospedaliero-Universitaria Senese, 53100 Siena, Italy

**Keywords:** CCDC78, sarcoplasmic reticulum, myopathy, centronuclear myopathy-4

## Abstract

*CCDC78* was identified as a novel candidate gene for autosomal dominant centronuclear myopathy-4 (CNM4) approximately ten years ago. However, to date, only one family has been described, and the function of CCDC78 remains unclear. Here, we analyze for the first time a family harboring a *CCDC78* nonsense mutation to better understand the role of CCDC78 in muscle. Methods: We conducted a comprehensive histopathological analysis on muscle biopsies, including immunofluorescent assays to detect multiple sarcoplasmic proteins. We examined *CCDC78* transcripts and protein using WB in *CCDC78*-mutated muscle tissue; these analyses were also performed on muscle, lymphocytes, and fibroblasts from healthy subjects. Subsequently, we conducted RT-qPCR and transcriptome profiling through RNA-seq to evaluate changes in gene expression associated with CCDC78 dysfunction in muscle. Lastly, coimmunoprecipitation (Co-Ip) assays and mass spectrometry (LC-MS/MS) studies were carried out on extracted muscle proteins from both healthy and mutated subjects. Results: The histopathological features in muscle showed novel histological hallmarks, which included areas of dilated and swollen sarcoplasmic reticulum (SR). We provided evidence of nonsense-mediated mRNA decay (NMD), identified the presence of novel *CCDC78* transcripts in muscle and lymphocytes, and identified 1035 muscular differentially expressed genes, including several involved in the SR. Through the Co-Ip assays and LC-MS/MS studies, we demonstrated that CCDC78 interacts with two key SR proteins: SERCA1 and CASQ1. We also observed interactions with MYH1, ACTN2, and ACTA1. Conclusions: Our findings provide insight, for the first time, into the interactors and possible role of CCDC78 in skeletal muscle, locating the protein in the SR. Furthermore, our data expand on the phenotype previously associated with *CCDC78* mutations, indicating potential histopathological hallmarks of the disease in human muscle. Based on our data, we can consider *CCDC78* as the causative gene for CNM4.

## 1. Introduction

Congenital inherited myopathies are a large and heterogeneous group of muscle diseases affecting skeletal muscle tissue [1,2,3]. They arise from genetically determined muscle protein defects and are classified based on muscle biopsy findings [2]. In the last few decades, advances in molecular genetics have allowed for the identification of an increasing number of causative genes linked to different types of hereditary myopathies [4]. It has become evident that mutations in the same gene can lead to more than one pathological and clinical phenotype and that the same pathological feature can result from mutations in different genes [5,6]. Some examples include mutations in *LMNA* [6], *SETX*, *SIGMAR1*, and *SLC5A7* [7,8,9,10].

The coiled-coil domain-containing protein 78 gene (*CCDC78*) was mapped by Daniels et al. [11] to chromosome 16p13.3. It encodes a protein that has been reported to be involved in centriole amplification in multiciliated cells [12,13] and in skeletal muscle function [14]. Majczenko et al. [14] found predominant gene expression in human skeletal muscle. They demonstrated, through immunostaining, a reticular pattern that partially overlapped the triad, the intersection of the T-tubule, and the terminal sarcoplasmic reticulum (SR). However, only one family with a *CCDC78* mutation has been reported to date [14]. The patients, harboring a splice-acceptor variant, exhibited early-onset distal muscle weakness, myalgia, and easy fatigue, despite having normal serum creatine phosphokinase (CPK) levels. A muscle biopsy revealed CCDC78 accumulation in large sarcoplasmic aggregates, which costained for desmin and actin and strongly for RyR1. CCDC78 localized to the sarcolemmal membrane and the perinuclear region as well as in a latticework-type pattern within the sarcoplasm. Costaining with RyR1 revealed that the latticework pattern corresponded to the triad, the intersection of the terminal SR, and the T-tubule. The authors thus suggested a possible interplay between RyR1 and CCDC78. Based on these findings, *CCDC78* was defined as a new candidate gene for autosomal dominant centronuclear myopathy-4 (CNM4; OMIM#614807). However, to date, the role of CCDC78 in muscle function remains unclear; additionally, the potential interaction of CCDC78 with RyR1 remains uncertain and yet to be demonstrated.

Here, we studied a family harboring the first ever reported nonsense mutation in the *CCDC78* gene. We performed optic and electron microscopy studies and multiple immunofluorescence assays on muscle biopsy to define possible histopathological patterns associated with CCDC78 dysfunction. We also analyzed *CCDC78* transcripts and protein from *CCDC78*-mutated muscle tissue; transcript and protein analyses were also carried out in muscle, lymphocytes, and fibroblast tissues from healthy subjects. Furthermore, we conducted RT-qPCR and transcriptome profiling through RNA-seq on mutated muscle tissues and some controls to evaluate differential gene expression changes related to CCDC78 dysfunction. Additionally, we explored whether this nonsense mutation acts through a loss-of-function or gain-of-function mechanism, as well as the potential activation of a non-mediated decay (NMD) mechanism. Lastly, following a co-immunoprecipitation (Co-Ip) assay and mass spectrometry analysis, we identified the main interacting proteins of CCDC78.

Our research provides insight, for the first time, into the interactors and possible role of CCDC78 in skeletal muscle. Furthermore, our data broaden the phenotype previously associated with *CCDC78* mutations, revealing potential histopathologic characteristics of disease in humans.

## 2. Materials and Methods

### 2.1. Standard Protocol Approvals and Patient Consents

Written informed consent was obtained from all patients involved in this study (see Table S1). All experiments described were approved by the local ethical committee (Regional Ethics Committee for Clinical Trials of the Tuscany Region, n. 17397, approval date 20 July 2020). All procedures were conducted in accordance with the Helsinki Declaration of 1975.

### 2.2. Morphological Analysis of Muscle Biopsies and HeLa Cells

Routine morphology and immunofluorescence analyses of muscle proteins were performed on biopsy samples of the right or left rectus femoris muscle according to standard protocols [15]. Ten-micrometer-thick sections were stained using standardized histological and histochemical methods including hematoxylin and eosin, reduced NADH, SDH, and COX. Additional details for ultrastructural studies can be found in the Supplementary Materials. For the immunofluorescence experiments, 8 μm thick sections were incubated with the following primary antibodies: anti-TRISK95 1:500 (kindly provided by Dr. Isabelle Marty, INSERM, Grenoble, France), anti-RyR 34C 1:500 (ThermoFisher Scientific), anti-CASQ1 rabbit antibody (ThermoFisher Scientific, Waltham, MA, USA), anti-CASQ1 mouse antibody (clone MA3-913; ThermoFisher Scientific), rabbit anti-CCDC78 (AV53233, Sigma-Aldrich, Burlington, MA, USA), rabbit anti-MYH1 (clone 1E15; Sigma-Aldrich), mouse anti-actin (alpha-Sarcomeric, clone 5C5, Sigma-Aldrich), and anti-RyR1 rabbit antibody, anti-SERCA1, and anti-desmin [16]. Cy2- or Cy3-conjugated anti mouse or anti rabbit secondary antibodies (Jackson ImmunoResearch Laboratories, Westgrove, PA, USA) were used for immunofluorescence detection. For the immunofluorescence experiments involving HeLa cells, we used the protocols already described by Genovese et al. [17] and Vorobjev et al. [18].

Morphometric analysis of muscle fiber cross-sectional area was estimated using Image J version n° 1.54 [19]. For measuring fiber cross-sectional area, the contrast of the micrographs was enhanced by 0.5%. The freehand tool was then used to mark individual fibers.

### 2.3. DNA Analysis

Total genomic DNA was extracted from peripheral blood samples with a MasterPure Complete DNA Purification Kit (Epicentre MasterPure DNA Purification Kit (cat#: MCD85201) according to the manufacturer’s instructions. DNA was analyzed using the whole-exome sequencing (WES) technique with NovaSeq6000 (Illumina, San Diego, CA, USA) on DNA extracted from peripheral blood samples (see Supplementary Information).

### 2.4. Cells Culture

Peripheral blood lymphocytes (PBLs) were obtained from the *CCDC78*-mutated patient and one control; mononuclear cells were separated by centrifugation on a Lymphoprep gradient [20]. The cells were washed twice in PBS, resuspended in RPMI 1640 medium with 10% fetal bovine serum (FBS), 1% l-glutamine, 1% penicillin/streptomycin, and 1% sodium pyruvate, and maintained at 37 °C with 5% CO_2_. To assess nonsense-mediated decay (NMD), PBLs were treated with cycloheximide (100 μg per milliliter), an inhibitor of nonsense-mediated decay, for 4 h. Both the PBLs from the patient and the control were collected after 4 h of culture for analysis.

Methods for primary fibroblast cultures and Hela cells [17] are described in detail in the Supplementary Information.

### 2.5. RNA Extraction

Total RNA, isolated from peripheral blood lymphocytes (PBLs), fibroblasts, and muscle tissue from *CCDC78*-mutated patient and controls, was processed using an RNeasy Mini Kit (Qiagen, Hilden, Germany) and QubitTM RNA IQ assay kit (Thermo Scientific, Waltham, MA, USA).

### 2.6. Transcripts Analysis

For each sample, 1 µg of total RNA was reverse transcribed using ImProm-II™ Reverse Transcriptase (Promega, Madison, WI, USA) and oligodT primers in a 20 µL volume. PCR amplifications were performed with primers surrounding the mutated region (Table S2). The products of individual PCR reactions were separated electrophoretically on agarose gels. After the purification of cDNA products with QIAquick PCR Purification kits (Qiagen, Hilden, Germany), sequencing was performed using the automated sequencer ABI 3500 (Applied Biosystems, Waltham, MA, USA). The results were analyzed using the Chromas software version 2.33 and compared with the reference sequence NG_032932.1.

### 2.7. Transcriptome Profiling

Transcriptome profiling by RNA-seq was conducted on three control muscle samples and the *CCDC78*-mutated patient using Illumina NovaSeq (San Diego, CA, USA), PE 2 × 150, via polyA selection (see Supplementary Information for details).

After extracting gene hit counts, the table of gene hit counts was used for downstream differential expression analysis. Reads per exon were grouped to calculate FPKM (Fragments Per Kilobase of exon model per Million mapped reads) values [21]. Only genes with FPKM > 1 were further analyzed. DESeq2, a well-established analysis tool [22,23], was used to compare gene expression between sample groups. The Wald test was employed to generate *p*-values and log2 fold changes. Genes with an adjusted *p*-value < 0.05 and an absolute log2 fold change > 1 were identified as differentially expressed genes for each comparison. Data quality and sample identity were assessed, with the original values normalized to account for factors like sequencing variations for accurate determination of differentially expressed genes (DEGs).

The significance of the set of commonly differentially expressed or spliced genes was evaluated using a non-parametric test. Gene Ontology (GO) enrichment analysis and pathway enrichment analysis were conducted on the statistically significant gene set using the GeneSCF v.1.1-p2 software. The goa_human GO list was used to cluster genes based on their biological processes and to determine their statistical significance, resulting in a list of genes by their gene ontologies.

### 2.8. RT-qPCR

RT-qPCR was performed using QuantiNova SYBR Green RT Mix (Qiagen, Hilden, Germany) according to the manufacturing instructions. The reaction mixture (total volume of 20 µL) contained 20 ng of RNA. All reactions were performed in triplicates on a CFX96 Real Time System (Bio-Rad, Hercules, CA, USA) (refer to Supplementary Information for details).

### 2.9. Western Blotting Study

Western blot (WB) analysis was used to evaluate CCDC78 and RyR1 expression in muscle tissue from the *CCDC78*-mutated patient, four heterozygous *RYR1*-mutated patients, and three controls. Notably, two *RYR1*-mutated patients harbored mutations located in the cytosolic shell (CS) of the protein (c.130C>T (p.Arg44Cys) and c.1654C>T (p.Arg552Trp), respectively), while the other two *RYR1*-mutated patients had mutations in the channel and activation core of RyR1 (c.11810C>T (p.Ser3937Leu) and c.11708G>A (p.Arg3903Gln), respectively), according to the protein’s well-known domains [24].

Muscle tissue (30–100 mg) was homogenized over ice using a Potter-type tissue homogenizer with Tissue Protein Extraction Reagent T-PER™ (Thermo Scientific, Rockford, IL, USA) and Complete Mini Anti-protease Cocktail Tablets (Roche Applied Science, Laval, QC, Canada) (see Supplementary Information for details).

The relative amounts of CCDC78 and RyR1 were analyzed by densitometry using the ImageJ software (“https://rsb.info.nih.gov/ij” accessed on 10 May 2023), with GAPDH used for normalization. When necessary, the antibody was stripped with Restore Western Blot Stripping Buffer (Thermo Scientific). Data represent the mean of five and more than two "independent replicates, respectively, for CCDC78 and RYR1 expression. Statistical analysis was performed by a two-tailed Student’s *t*-test, with *p*-values < 0.05 and 0.01 considered statistically significant and highly significant, respectively.

### 2.10. Co-Immunoprecipitation (Co-Ip) Assay

Fifty milligrams of muscle tissue from healthy controls and the *CCDC78* mutated patient were lysed over ice using a Potter-type tissue homogenizer with IP Lysis/Wash Buffer (Thermo Scientific, Rockford, IL, USA) and Complete Mini Anti-protease Cocktail Tablets (Roche Applied Science, Laval, PQ, Canada) according to the manufacturer’s’ instructions. The Co-Ip assay was performed using a Pierce Crosslink Magnetic IP/Co-Ip Kit (Thermo Scientific, Rockford, IL, USA). Anti-CCDC78 (AV53233, Sigma-Aldrich) was diluted to a final concentration of 10 µg/100 µL. Co-Ip was carried out using disuccinimidyl suberate (DSS) for crosslinking antibodies to beads covalently. The immunoprecipitated protein was loaded into 5%, 12%, and 4–15% gels. The gel was fixed with gentle agitation and then it was stained (staining solution: 0.1% Coomassie Brilliant Blue R-250, 50% methanol, and 10% glacial acetic acid). Afterwards, the gel was destained and stored for mass spectrometry analysis. For each immunoprecipitation assay, positive controls of protein expression at the whole lysate before Co-IP and negative controls with nonspecific IgG (111-035-045, Jackson ImmunoResearch Laboratories, West Grove, PA, USA) were performed. GAPDH was used as a negative control to demonstrate the specificity of Co-Ip (refer to Supplementary Information for details).

### 2.11. Mass Spectroscopy Study

The blue-Coomassie-stained 1D gel was used for protein analysis. Seven bands of interest were selected and manually excised from the gel for mass spectrometric analysis. Each band of interest underwent destaining as well as successive reduction, alkylation, and enzymatic digestion [25] (see Supplementary Information). The final solution of the digested proteins was used for the mass spectrometry experiments.

### 2.12. nLC-nESI-HRMS/MS

The enzymatically digested solution obtained from the gel band was analyzed by nano liquid chromatography (nLC) coupled with high-resolution mass spectrometry (HRMS) equipped with a nanoelectrospray (nESI) interface (nLC-nESI-HRMS/MS) [26]. The instrument was composed of a nanoLC system EASY-nLC 1200 connected to an LTQ Orbitrap hybrid mass spectrometer (Thermo Scientific, Bremen, Germany) (see Supplementary Information for details).

### 2.13. Filtering and Selecting Interactors Candidates for Validation

The acquired data were analyzed using the Mascot 2.4 search engine (Matrix Science Ltd., London, UK) against a human database created from NCBI. Searches were performed allowing: (i) trypsin as the enzyme, (ii) up to two missed cleavage sites, (iii) 10 ppm of tolerance for the monoisotopic precursor ion and 0.5 mass unit for monoisotopic fragment ions, and (iv) carbamidomethylation of cysteine and oxidation of methionine as variable modifications. A target–decoy search was used as follows: a false discovery rate (FDR) of 1% was imposed, and the criterion used to accept protein identification included a probabilistic score sorted by the software. We identified a total of 478 known database entries after eliminating protein contaminants such as the keratin family and immunoglobulin to obtain 367 total interactors with a number of unique peptides per protein ≥ 1.

### 2.14. Three-Dimensional (3D) Modelling

Three-dimensional (3D) modelling was conducted using SwissModel (https://swissmodel.expasy.org/” accessed on 10 November 2023) to illustrate the potential impact of the mutation on the CCDC78 protein structure. The analysis utilized the human template A2IDD5.1.A (GMQE = 0.66) for the 48 kDa isoform, and for the 52 kDa isoform, the software employed the near-atomic resolution structure of the highly similar (70%) S9XHM5.1.A template from Camelus ferus (GMQE = 0.69). Moreover, using the DAS method (https://tmdas.bioinfo.se/ accessed on 5 November 2023) [27], we analyzed the potential presence of transmembrane domains in the CCDC78 protein. Curves were generated through pairwise comparison of proteins in the test set in an “each against the rest” manner. The plots displayed two cutoffs: a “strict” one at a 2.2 DAS score, and a “loose” one at 1.7. The hit at 2.2 provided information on the number of matching segments, while a hit at 1.7 indicated the actual location of the transmembrane segment. Project HOPE, a web server that investigates the structural consequences of variants by cooperating with UniProt and DAS prediction algorithms [28], was utilized to study the p.Glu404Lys variant in the NM_001378030.1 isoform.

## 3. Results

### 3.1. The Nonsense CCDC78 Mutation Leads to a Dystrophic Muscle Process Mainly Involving the SR

**Figure 1:**
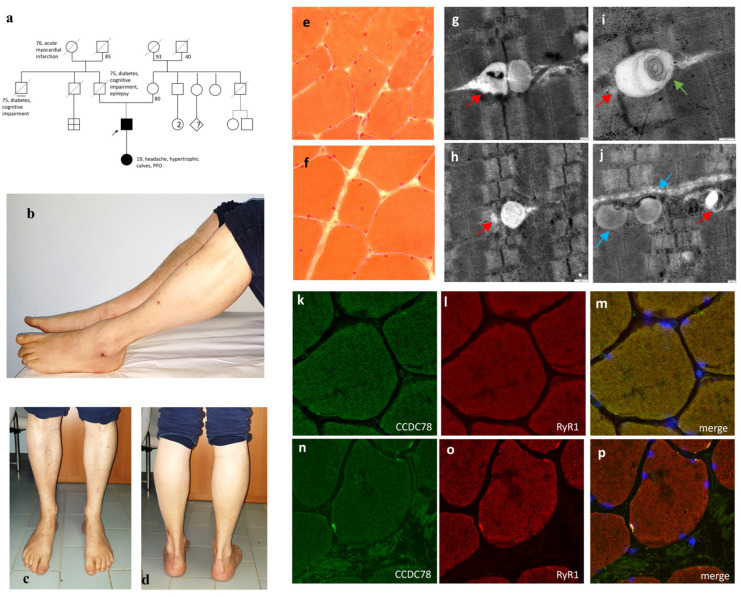
Pedigree of the *CCDC78*-mutated patients (**a**), photographs of the proband showing slight hypertrophy of the calves bilaterally (**b**–**d**), muscle biopsy with E-H staining (20X) indicating nuclear centralizations (**e**,**f**), and TEM (scale bar = 200 nm) revealing peculiar dilated terminal SR (red arrows), whirls of redundant membranes (green arrow), and areas of dilated and swollen SR with numerous abnormal accumulations of membranous material (blue arrows) (**g–j**). Immunofluorescent analysis on muscle tissue (20X): by comparing control (**k**–**m**) and *CCDC78*-mutated muscles (**n**–**p**) after CCDC78 (**k**,**n**) and RyR1 staining (**l**,**o**), we showed CCDC78 aggregates, overlapping with RyR1.

Our patient, a 52-year-old male, has been experiencing myalgias since the age of about 40. He has continuous painful muscle cramps in the calf and foot muscles, accompanied by excessive sweating and easy fatigue. Blood chemistry results showed persistent CPK elevation, with the last two CPK measurements being 655 and 663 U.I./L, respectively; myoglobin levels were found to be at 96 ng/mL. Aspartate aminotransferase and alanine aminotransferase levels were measured at 36 I.U./L and 56 I.U./L, respectively. A liver ultrasound revealed mild hepatic steatosis. Neurological examination revealed lively deep tendon reflexes in the lower limbs and slight hypertrophy of the calves bilaterally (Figure 1b–d) without any strength deficit. An EMG study conducted at 50 years of age was normal. The patient’s father, deceased at 80 years of age, was reported to have had senile dementia, epilepsy, and bilateral calf hypertrophy. The patient’s daughter was found to have a patent foramen ovale and bilateral calf hypertrophy (Figure 1a).

**Figure 2:**
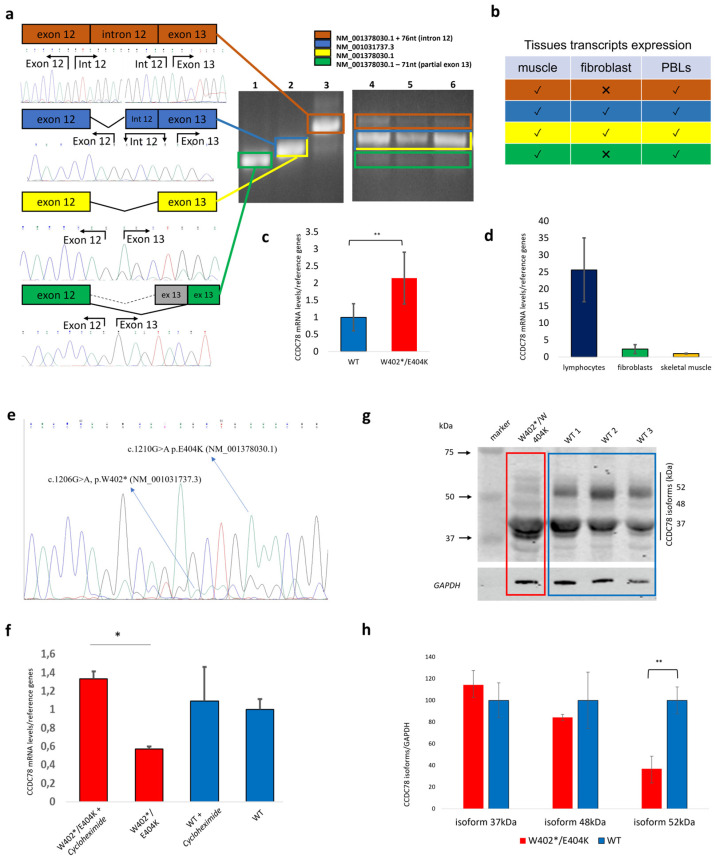
PCR amplification of *CCDC78* exons 10–14 showed the presence of four transcripts in healthy controls (**a**). Agarose gel electrophoresis (2% agarose) of PCR-amplified products using specific PCR primer sets. Lanes 1–3 display the sequenced PCR products in control muscle; lanes 4, 5, and 6 show the transcript pattern of exon regions 10–14 in PBLs, muscle, and fibroblasts from controls, respectively. Tissues transcripts in muscle, fibroblast, and blood from controls are shown (v = transcript sequenced, X = transcript not sequenced (**b**). RT-qPCR in muscle showed a significant increase in our patient (**c**) calculated by setting the ratio of *CCDC78*/reference genes expression in the control group to 1. Relative expression levels were calculated relative to *HPRT1*, *SERPINC1*, and *ZNF80* mRNA levels. The bars show the mean ± SD (n = 2, ** *p* < 0.01). RT-qPCR showed the relative expression levels of *CCDC78* in lymphocytes, fibroblasts, and muscle of control samples (**d**). *CCDC78* expression in muscle tissue was set to 1 (SD ± 0.09), and the relative expressions in fibroblasts and lymphocytes were, respectively, 2.3 (SD ± 1.30045) and 25.62 (SD ± 9.39). Relative expression levels were calculated in relation to *HPRT1* and *ZNF80* mRNA levels (n = 2). To assess NMD due to the p.W402* mutation, as suggested by an apparently lower level of transcript NM_001031737.3 from muscle sample (**e**), we analyzed the *CCDC78* expression in lymphocytes of the *CCDC78*-mutated patient and control subjects in basal conditions and after cycloheximide treatment. The patient showed a significant increase in transcript values compared to the control (**f**). Relative expression levels were calculated relative to *HPRT1*, *SERPINC1*, and *ZNF80* mRNA levels and set to 1. (n = 3, *p* < 0.01). Western blot (WB) analysis showing the expression levels of CCDC78 isoforms in muscle tissue of patient and three controls (**g**). The bar graph (**h**) shows the isoform expression fold change in CCDC78, calculated by setting the ratio of CCDC78 protein/GAPDH protein band intensities in the control group to 100. The bars show the mean ± SD. (n ≥ 5, * *p* < 0.01).

The WES revealed a heterozygous mutation c.1206G>A (p.Trp402*) in our patient, located in exon 13 of the *CCDC78* gene (rs752371476; NM_001031737.3). This mutation results in a premature stop codon at position 402 out of 438. There are two known alternative *CCDC78* transcripts: NM_001378030.1 and NM_001031737.3. The NM_001378030.1 transcript (shown in yellow in Figure 2a) is 1611 bp in length and encodes the 438 aa protein isoform. The NM_001031737.3 transcript (shown in blue in Figure 2a) is 1585 bp in length and, unlike the previous one, contains four nucleotides (GCAG) at the beginning of exon 13 and codes for the 470 aa protein isoform.

The identified *CCDC78* variant was predicted to be damaging by the CADD-phred prediction tools (CADD-phred = 44) and BayesDel algorithm (addAF score 0.5116, noAF score 0.5125). The Genomic Evolutionary Rate Profiling (GERP) score was 1.96, thus indicating that the variant may be under evolutionary constraint. The minor allele frequency (MAF) was <0.01%, the ExAc all frequency of the variant was 0.00001671%, and the GnomAD all was 8.01218e-06. Notably, the mutation resulted as a missense variant in the longer isoform of the transcript: c.1210G>A (p.Glu404Lys, NM_001378030.1). The variant was Sanger-confirmed (Figure S2). Variant segregation analysis in the proband’s mother and daughter found the mutation only in the daughter. The proband’s father was deceased and not testable.

The muscle biopsy from the left vastus lateralis muscle showed heterogeneity in the caliber and shape of the muscle fibers, with both hypotrophic and hypertrophic fibers present. There were no signs of cellular necrosis or inflammatory infiltrates. Nuclear centralizations were observed in 6–7% of muscle fibers (Figure 1e,f). Gomori’s trichrome stain and oxidative reactions were normal. No increase in PAS-positive or sudanophilic material was detected. A predominance of type 2 fibers was evident. Dystrophin epitope immunohistochemical staining revealed a normal expression of the sarcolemmal C-terminal (dystrophin 2) and N-terminal domain (dystrophin 3). The immunohistochemical study with antibodies for caveolin, dysferlin, 35 kDa delta-sarcoglycan, 35 kDa gamma-sarcoglycan, 43 kDa beta-sarcoglycan, and 50 kDa alpha-sarcoglycan antibodies showed normal results. Transmission electron microscopy (TEM) revealed a dilated terminal SR, whirls of redundant membranes, and areas of dilated and swollen SR with numerous areas of abnormal accumulations of membranous material (Figure 1g–j). Immunofluorescent analysis on muscle tissue showed CCDC78 aggregates, overlapping with RyR1 (Figure 1k–p) and desmin.

### 3.2. Human Tissues Express Multiple CCDC78 Isoforms, and the Nonsense CCDC78 Variant Leads to a Mutant Allele That Is Mostly Degraded by NMD

Several PCRs including exons 10–14 were set up to study the 3′ region of the transcript where the identified *CCDC78* mutation is located. PCRs were performed on RNA extracted from fibroblasts, lymphocytes, and muscle tissues derived from controls. We identified both NM_001031737.3 and NM_001378030.1 transcripts. Moreover, we also detected two additional in-frame transcripts due to alternative splicing involving the last exons of the gene: the first transcript was derived from the retention of intron 12 (NM_001378030.1:r.1201_1275del), and the second transcript was derived from the activation of a new acceptor site in exon 13 (NM_001378030.1:r.1201_1275del) (Figure 2a,b).

To determine if the stop codon leads to NMD, as suggested by an apparent decrease in NM_001031737.3 transcript levels (Figure 2e), we analyzed *CCDC78* expression in the patient’s lymphocytes compared to a control subject. We compared gene expression under basal conditions and after treatment with cycloheximide, a known NMD inhibitor. The patient exhibited a significant increase in transcript levels post-treatment compared to the control (n = 3, *p* < 0.01) (Figure 2e,f), indicating NMD activation.

CCDC78 expression analysis by WB in the *CCDC78*-mutated patient and three controls showed the presence of different protein isoforms at 52 kDa (Uniprot: H3BLT8), 48 kDa (Uniprot: A2IDD5-1), and 37 kDa (Uniprot: A2IDD5-5) (Figure 2g). Only the 52 kDa isoform in the *CCDC78*-mutated patient was significantly reduced compared to the controls (36.85 ± 11.61%, *p*-value < 0.01). The 48 and 37 kDa CCDC78 isoforms were, respectively, 84.33 ± 2.75% (SD) and 114.28 ± 2.75% (SD) compared to the controls (Figure 2h).

*CCDC78* gene expression was evaluated by RT-qPCR in muscle, showing a significant increase in expression in our patient compared to the healthy controls (*p* < 0.01) (Figure 2c). Moreover, the analysis of relative expression of *CCDC78* in lymphocytes, fibroblasts, and muscle tissue from the healthy controls revealed a lower *CCDC78* expression in muscle tissue. By setting muscle tissue expression to 1 (SD ± 0.09), the relative expressions in fibroblasts and lymphocytes were 2.30 (SD ± 1.30) and 25.62 (SD ± 9.38), respectively (Figure 2d). Accordingly, *CCDC78* was found to be very lowly expressed in muscle tissue and was not detectable by the RNA-seq analysis we performed. These findings are in line with previous data from Gonorazky et al. [29], which showed in GTEx skeletal-muscle controls and in a large cohort of muscle biopsies 15 genes, including *CCDC78* expressed at <1 RPKM. Therefore, we can speculate that the good protein expression in muscle could possibly be due to a very high stability of *CCDC78* transcripts in cells.

**Figure 3:**
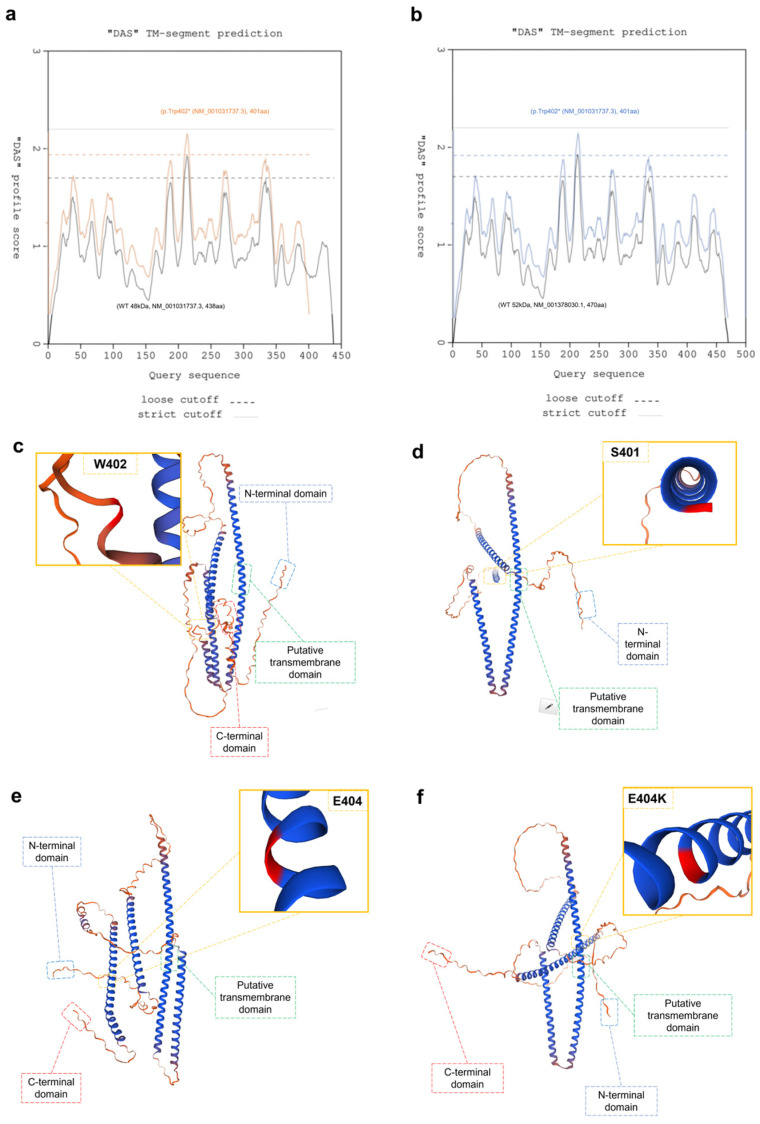
Curves obtained through pairwise comparison of the proteins in the test set in an “each against the rest” fashion (**a**,**b**): there are two cutoffs indicated on the plots: a “strict” one at 2.2 DAS score, and a “loose” one at 1.7. The hit at 2.2 is informative in terms of the number of matching segments, while the hit at 1.7 gives the actual location of the transmembrane segment (TS). The segments reported in the feature table (FT) records of the SwissProt database are marked at 1.0 DAS score (“FT lines”). In (**a**), we report a staggered superposition of the potential TS predictions for both the WT 48 kDa protein (NM_001031737.3) (black lines) and the relative mutated protein (p.Trp402* (NM_001031737.3)) (orange lines): WT TS starts at 209 and stops at 216 (length ~8, cutoff ~1.7); in the mutated protein, TS starts at 210 and stops at 216 (length ~7, cutoff ~1.7). The plots are very similar; however, the TS is shorter in the mutated protein. In (**b**), we report a staggered superposition of the potential TS predictions for both the WT 52kDa protein (NM_001378030.1) (black lines) and the relative mutated protein (p.Glu404Lys (NM_001378030.1)) (blue lines): both WT and mutated TS start at 209 and stops at 216 (length ~8, cutoff ~1.7). The plots are identical and perfectly overlapping. Three-dimensional modelling: both the WT isoforms and the relative mutated protein are shown (A2IDD5_CCDC78_HUMAN, 438aa, WT (**c**), A2IDD5_CCDC78_HUMAN, 401aa, p.Trp402* (**d**), H3BLT8_CCDC78_HUMAN, 470aa, WT. (**e**), H3BLT8_CCDC78_HUMAN, 470aa, p.Glu404Lys (**f**)). For both the variants, we observed a different spatial orientation of the first (Glu56-Asp105) and the fourth (Asp381-Ser401) alpha-helices with respect to the second one (Asn156-Asp259).

Through the Dense Alignment Surface (DAS) method [27], we predicted possible transmembrane segments (TS) for the CCDC78 protein. In the wild type (WT) 48 kDa, WT 52 kDa, and mutated 52 kDa isoforms, the potential TS was located between aminoacidic positions 209 and 216. Only the mutated 48 kDa isoform showed a potential TS between positions 210 and 216 (Figure 3). To define the possible structural effects of the variants, 3D models of wild-type human CCDC78 (both NM_001031737.3 and NM_001378030.1) and its variants (p.Trp402*, NM_001031737.3 and p.Glu404Lys, NM_001378030.1) were generated. Our data showed a critical change in the 3D structure driven by both p.Trp402* (NM_001031737.3) and p.Glu404Lys (NM_001378030.1). Other than a reduced protein length for p.Trp402*, as expected, we observed, for both the variants, a different spatial orientation of the first (Glu56-Asp105) and fourth (Asp381-Ser401) alpha-helixes with respect to the second one (Asn156-Asp259), which likely contains the predicted TS (Figure 3). This effect may lead to a dysfunctional CCDC78 protein. Furthermore, the analysis of the mutated 52 kDa isoform by HOPE (“https://www3.cmbi.umcn.nl/hope/” accessed on 10 May 2023) indicated that the Lys404 aminoacidic residue, larger than the wild-type Glu404 residue, might lead to bumps on the 3D structure of the protein near a highly conserved position of CCDC78, thus suggesting a possibly damaging effect. Moreover, the Glu > Lys substitution, swapping a negative to positive residue, may further affect protein function trough repulsion of other residues with the same charge. We also noted a noticeable movement in the N-terminal domain caused by the p.Trp402* variant (Figure 3c,d); this effect could potentially alter co-translational and post-translational modifications in the protein, thereby affecting protein stability, localization, or activity.

### 3.3. The Dysfunction of CCDC78 Leads to an Upregulation of Genes Involved in the SR

We performed a comprehensive RNA-seq analysis using muscle tissue from the *CCDC78*-mutated patient and three controls to examine changes in gene expression associated with CCDC78 dysfunction. The box plot analysis provides a visual representation of the raw (Figure S1a) and normalized (Figure S1b) expression values. Figure S1c shows the distances measured using expression values from each sample, where the shorter the distance, the more closely related the samples were. This method allowed us to determine the relatedness of the two groups; Euclidean distance analysis followed by hierarchical clustering analysis indicated that each set of replicates clustered together. Principal component analysis (PCA) was performed on the covariance matrix of 14,858 genes expressed in muscle tissue across our four samples. PCA revealed differences in gene expression between the two experimental conditions, confirming that the control replicates clustered together well and that gene expression was mainly separated by health condition (PC1, 60% variance) (Figure S1d).

**Figure 4:**
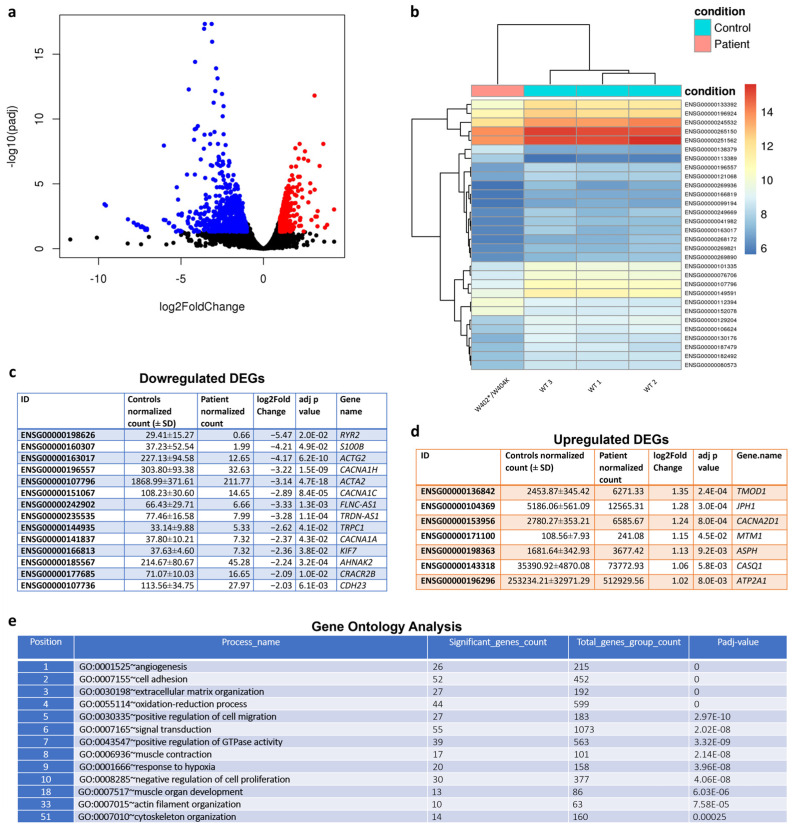
Volcano plots showing gene expression differences between the *CCDC78* mutated patient and controls (**a**). The plot shows the global transcriptional change across the groups compared. All the genes are plotted, and each data point represents a gene. The log2 fold change in each gene is represented on the x-axis, and the log10 of its adjusted *p*-value is on the y-axis. Genes with an adjusted *p*-value less than 0.05 and a log2 fold change greater than 1 are indicated by red dots and represent upregulated genes. Genes with an adjusted *p*-value less than 0.05 and a log2 fold change less than −1 are indicated by blue dots and represent downregulated genes. Hierarchical clustering analysis of the *CCDC78* mutated patients and controls with heatmap density color representation of differentially expressed genes (DEGs) (**b**). This analysis was performed to visualize the expression profile of the top 30 genes sorted by their adjusted *p*-values. This analysis is useful to identify co-regulated genes across the conditions. In (**c**,**d**), a series of downregulated and upregulated genes associated with muscular function and SR are reported. Normalized counts for each gene in the controls and the *CCDC78* mutated patient are reported. Gene ontology (GO) analysis (**e**): top GO terms of genes associated with differentially expressed transcripts identified in muscles from the *CCDC78*-mutated patient as compared to controls. *p*-values were adjusted by false discovery rate (FDR) multiple testing correction.

We identified 1035 DEGs in muscle tissue between our patient and the controls (Figure 4a). Hierarchical clustering analysis of gene expression data confirmed a highly similar expression profile, with the controls clustering together apart from the *CCDC78* mutated patient (Figure 4b).

To understand the biological functions of the identified *CCDC78* mutated-patient-associated DEGs, we performed a functional gene enrichment analysis. In the discovery set, we observed a statistically significant association with various biological processes. Among them, genes associated with “extracellular matrix organization” have been previously shown to be differentially expressed in other types of genetic myopathy [30] and appear to be common to many dystrophic processes. The top 10 processes sorted by their adjusted p-values were “angiogenesis”, “cell adhesion”, “extracellular matrix organization”, “oxidation-reduction process”, “positive regulation of cell migration”, “signal transduction”, “positive regulation of GTPase activity”, “muscle contraction”, “response to hypoxia”, and “negative regulation of cell proliferation” (Figure 4e). Interestingly, among all the 411 statistically significant processes sorted by their adjusted *p*-values and most relevant to a muscle disease, we found the following: “muscle contraction” (8th position), “muscle organ development” (18th), “actin filament organization” (33rd), “cytoskeleton organization” (51st), “calcium ion transport into cytosol” (63rd), “response to muscle activity” (72nd), “actin cytoskeleton organization” (79th), “detection of calcium ion” (110th), “actomyosin structure organization” (136th), “calcium ion transport” (180th), “clustering of voltage-gated sodium channels” (183rd), “endoplasmic reticulum organization” (268th), “actin cytoskeleton reorganization” (287th), “actin filament bundle assembly” (292nd), “membrane depolarization during action potential” (293rd), “calcium ion transmembrane transport via high voltage-gated calcium channel” (317th), “negative regulation of skeletal muscle cell differentiation” (321st), and “calcium ion import” (374th).

Among the 1035 muscular DEGs, we found the upregulation of a series of genes involved in the SR and excitation–contraction coupling (ECC) with a log2 fold change ranging from 1.02 to 1.35: *TMOD1*, *JPH1*, *CACNA2D1*, *MTM1*, *ASPH*, *CASQ1,* and *ATP2A1* (Figure 4d). The other top upregulated DEGs included the following: *PKP2*, *DBNDD1*, *ARHGAP36*, *MSTN*, *MYLK2*, *SGCD*, *TTL*, *VEZT*, *WHAMM*, *SEMA4D*, *NIN* and *PLXNA1*. The top downregulated DEGs included: *S100B*, *ACTG2 CACNA1H*, *ACTA2*, *CACNA1C*, *FLNC-AS1*, *TRDN-AS1*, *TRPC1*, *CACNA1A*, *KIF7*, *AHNAK2*, *CRACR2B,* and *CDH23* (Figure 4c).

### 3.4. CCDC78 Interacts with Two Important SR Proteins: SERCA1 and CASQ1

**Figure 5:**
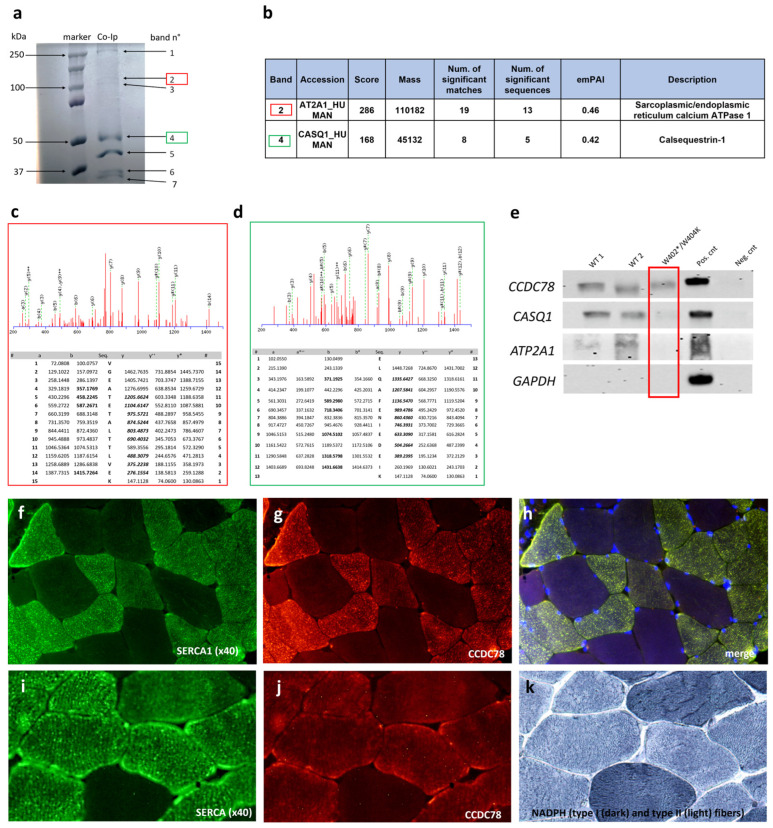
Co-immunoprecipitation (Co-Ip) of CCDC78-interacting proteins (**a**): muscle lysates were subjected to Co-Ip using anti-CCDC78 antibody and analyzed by SDS-PAGE followed by blue Coomassie staining. Two bands with molecular weights of around 50 and 110 kDa were detected (band 2, in red, and 4, in green). Lane 1, protein molecular weight ladder; lane 2, muscle lysate of healthy control. Identification of CCDC78-interacting proteins by nLC-nESI-HRMS/MS (**b**). Mass spectrometry (MS) analysis of band 2 (**c**): fragmentation spectra of 781.42053 m/z identifying AT2A1_HUMAN. The corresponding putative amino acid sequences were taken from MASCOT search. The VGEATETALTTLVEK producing an Ions Score of 52 (expect: 5.9 × 10^−5^). The m/z values of detected positive ion fragments are in red. The ‘b’ and ‘y’ ions are singly charged fragments (molecule + 1 H +) produced by fragmentation from the N- and C-terminus, respectively; ‘b ++’ and ‘y ++’ ions are the corresponding doubly charged fragments (molecule + 2 H +). The y* ions are y ions with loss of water. MS analysis of band 4 (**d**): fragmentation spectra of 789.38879 m/z identifying CASQ1_HUMAN and the corresponding putative amino acid sequences taken from MASCOT search. The ELQAFENIEDEIK producing an Ions Score of 47 (expect: 3.9 × 10^−5^). Western blot analysis (**e**). Proteins immunoprecipitated using CCDC78 antibody were immunoblotted on membranes using anti-SERCA1 and anti-CASQ1 antibodies. SERCA1 and CASQ1 were detected in wild-type pulldowns (WT1, WT2) but not in *CCDC78*-mutated patient pulldown (PT). ATP2A1 and CASQ1 were also detected in the total cell lysate (+Cnt) but not in the IgG control (−Cnt.). Membranes were immunoblotted with anti-GAPDH (negative Co-IP control) and anti-CCDC78 (positive Co-IP control). Colocalization between SERCA1 and CCDC78 in control muscle tissue (**f**–**h**). In the muscle seriated sections, we found a colocalization of CCDC78, SERCA1, and NADPH diaphorase (**i**–**k**).

Our Co-Ip assays clearly showed the presence of several bands (Figure 5a), each corresponding to a potentially different CCDC78-interacting protein. A band located between 37 and 50 kDa corresponding to CCDC78 was identified in the CCDC78 pulldown lane or input lane but not in the IgG control lane on the blue Coomassie gel and WB; this finding demonstrates that CCDC78 can be efficiently and specifically immunoprecipitated from the muscle tissue extract. In the blue Coomassie gel, we identified a band corresponding to ~110 kDa (band 2) and a ~50 kDa band (band 4); they were manually excised from the gel for the nLC-nESI-HRMS/MS analysis, as described in the Materials and Methods section. From band 2, applying the filtration criteria described in the experimental procedure and considering a significance threshold of *p* < 0.05, we were able to identify 60 database entries. By filtering by the total score, emPAI, and mass ≥ 100 kDa and ≤120 kDa, we were able to identify sarco-endoplasmic reticulum calcium ATPase1 (SERCA1), having a sequence coverage of 28% (Figure 5b,c, Table S3). Band 4 was analyzed according to the same procedure for band 2; we thus identified 111 database entries, and, among these, we selected calsequestrin1 (CASQ1), having a sequence coverage of 11% (Figure 5b–d). We then confirmed these findings by WB analysis. Notably, the presence of the *CCDC78* mutation was able to abolish the interactions with SERCA1 and apparently to decrease the interaction with CASQ1 (Figure 5e). Our results were corroborated by our transcriptome data, which showed an upregulation of both *ATP2A1* and *CASQ1* in our *CCDC78*-mutated patient compared to the controls (Figure 4d).

**Figure 6:**
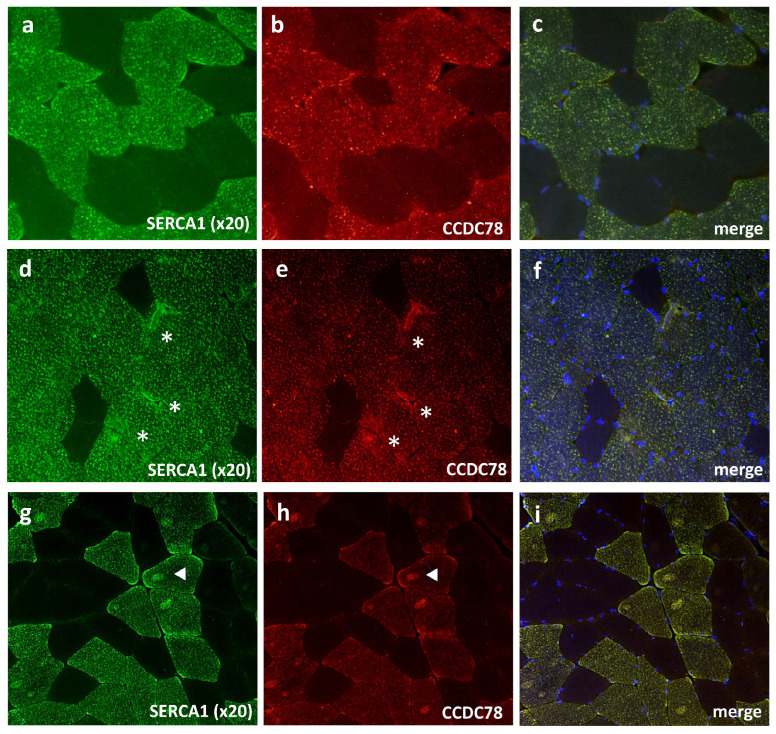
SERCA1 and CCDC78 aggregates (asterisks) in CCDC78-mutated muscle (**d**–**f**) compared to control (**a**–**c**). RYR1-mutated muscle (**g**–**i**): costaining of SERCA1 and CCDC78 with cores (arrow heads).

The immunofluorescence study in muscle tissue was highly consistent with the interactions of the proteins found; we indeed observed a strong colocalization between CCDC78 and SERCA1 (Figure 5g,h) in the control muscle tissue. Notably, in the histological serial sections, we found a colocalization of CCDC78, SERCA1, and NADPH diaphorase, thus confirming the predominant localization of CCDC78 in type II fibers (Figure 5i–k). In the *CCDC78*-mutated muscle, some SERCA1 (Figure 6) and CASQ1 aggregates were evident. Notably, in our *RYR1*-mutated patients showing central cores, we observed a costaining of SERCA1, CCDC78 (Figure 6g–i), and CASQ1 aggregates at the cores.

### 3.5. CCDC78 Interacts with Other Proteins outside the SR: MYH1, ACTN2, and ACTA1

**Figure 7:**
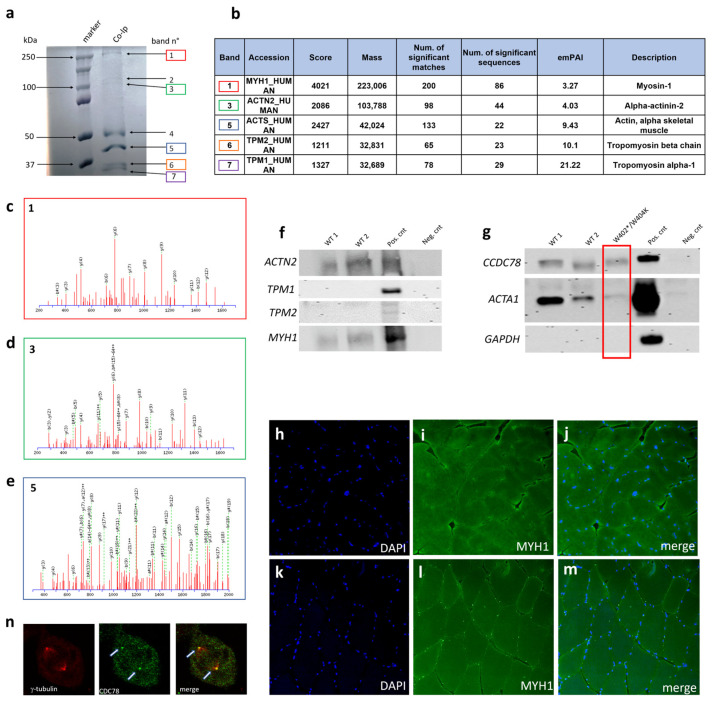
Co-Ip of CCDC78-interacting proteins (**a**). Five bands were further detected: ~250 kDa (band 1, in red), ~100 kDa (band 3, in green), ~45 kDa (band 5, in blue), and two bands in the 25–37 kDa range (band 6, in orange, band 7, in purple). Lane 1, protein molecular weight ladder; lane 2, muscle lysate of healthy patient. Identification of CCDC78-interacting proteins by nLC-nESI-HRMS/MS (**b**). Mass spectrometry (MS) analysis of band 1 (**c**): fragmentation spectra of 859.91791 m/z identifying MYH1_HUMAN. The LQNEVEDLMIDVER sequence produced an Ions Score of 81 (expect: 6.7 × 10^−8^). Mass spectrometric analysis of band 3 (**d**): fragmentation spectra of 908.42621 m/z identifying ACTN2_HUMAN. The ISSSNPYSTVTMDELR sequence produced an Ions Score of 82 (expect: 2.1 × 10^−8^). Mass spectrometric analysis of band 5 (**e**): fragmentation spectra of 1276.58142 m/z identifying ACTS_HUMAN. The LCYVALDFENEMATAASSSSLEK sequence produced an Ions Score of 77 (expect: 5.6 × 10^−8^). Western blot analysis (**f**,**g**). Proteins immunoprecipitated using CCDC78 antibody were immunoblotted using anti-ACTN2, anti-MYH1, anti-ACTA1, anti-TPM1, and anti-TPM2 antibodies. ACTN2, MYH1, and ACTA1 were detected in wild-type pulldowns (WT1, WT2). ACTN2, MYH1, and ACTA1 were also detected in the total cell lysate (CTR+) but not in the IgG control (CTR−). Membranes immunoblotted with anti-TPM1 and anti-TPM2 detected protein only in the total cell lysate (CTR+) but not in the WT muscles and IgG control (CTR−). Membranes were immunoblotted with anti-GAPDH (negative Co-IP control) and anti-CCDC78 (positive Co-IP control). MYH1 staining in control (**h**–**j**) and *CCDC78*-mutated (**k**–**m**) muscles (20×): perinuclear small MYH1 aggregates were present in mutated muscle. HeLa cells showing a costaining of CCDC78 and gamma-tubulin (**n**).

In the blue Coomassie gel, we identified three additional bands corresponding to ~250 kDa (band 1), ~100 kDa (band 3), and 37–50 kDa (band 5), respectively. These bands underwent the same procedures as above for nLC-nESI-HRMS/MS analysis. From band 1, we were able to identify 168 database entries. After filtering analysis, we selected MYH1, with a sequence coverage of 58% (Figure 7a–c, Table S3). For band 3 and 5, we identified 73 and 56 database entries, respectively. Among these, we selected ACTN2, with a sequence coverage of 39%, and ACTA1, with a sequence coverage of 63% (Figure 7a,b,d). We then confirmed by WB the interaction of CCDC78 with MYH1, ACTN2, and ACTA1 in the analyzed muscle samples. Once again, the *CCDC78* mutation appeared to decrease the interaction between CCDC78 and ACTA1 (Figure 7f,g).

The nLC-nESI-HRMS/MS analysis of bands 6 and 7 (Figure 7a) identified 70 and 68 entries, respectively. By filtering by the total score, emPAI, and mass between 25 kDa and 37 kDa, we selected TPM1 and TPM2 as potential interactors (Figure 7b). However, WB did not confirm any further possible interactions (Figure 7f).

Our immunofluorescent studies in muscle tissue showed perinuclear small MYH1 aggregates (Figure 7h–m) and a small number of ACTA1 aggregates in our *CCDC78*-mutated patient compared to the controls. In addition to the selected proteins, we examined the possible colocalization of CCDC78 and gamma-tubulin in HeLa cells, considering the previous association of CCDC78 with centriolar structures. We observed the localization of both proteins at the spindle pole (Figure 7n), the region of the cell where the centrosome is located, supporting a role of CCDC78 in centrioles.

### 3.6. CCDC78 Does Not Directly Interact with RyR1

**Figure 8:**
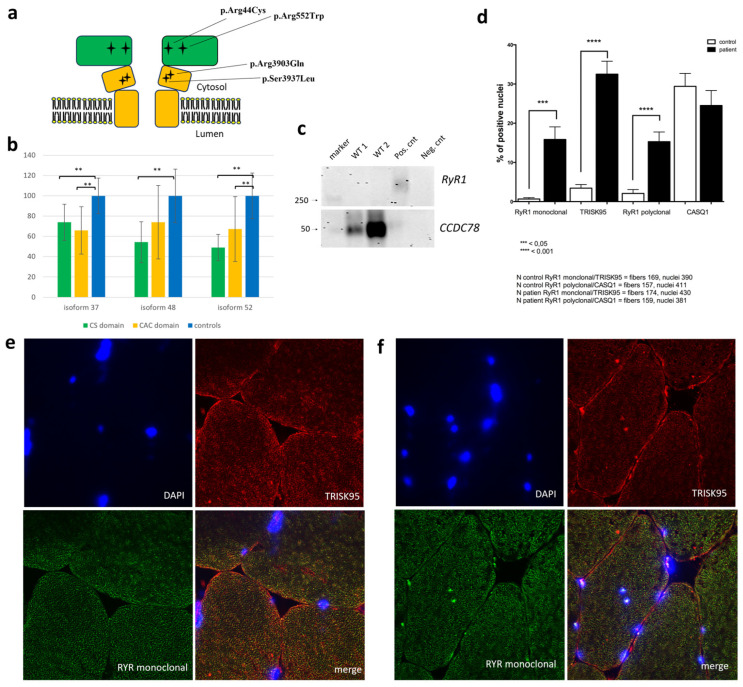
Schematic representation of cytosolic shell (CS, in green) and channel and activation core (CAC, in yellow) of RyR1 (**a**): black star signs represent the analyzed *RYR1* mutations. CCDC78 expression analysis by WB in RYR1-mutated patients (n = 4) compared to controls (n = 3) (**b**): significant reduction (**, *p* < 0.01) in 37 kDa, 48 kDa, and 52 kDa isoforms compared to controls. The two *RYR1*-mutated patients harboring a mutation in the CS showed a significant reduction in 37, 48, and 52 kDa isoforms compared to controls (*p* < 0.01); the patients carrying a *RYR1* mutation in CAC showed a significant reduction only for 37 and 52 kDa isoforms. Western blot analysis (**c**): Proteins immunoprecipitated using CCDC78 antibody were immunoblotted using anti-RyR1 and anti-CCDC78 antibodies. CCDC78 was detected in wild-type pulldowns (WT1, WT2), in the total cell lysate (positive control) but not in the IgG control (negative control). Membranes immunoblotted with anti-RyR1 detected the protein only in the total cell lysate. Morphometric analysis of muscle fiber cross-sectional area (**d**): in *CCDC78*-mutated muscle, we observed a significant increase in %positive RyR1 and Trisk95 nuclei compared to controls. RyR1, DAPI, and Trisk95 staining in the *CCDC78*-mutated muscle (**f**) and control (**e**): in mutated muscle, RyR1 aggregates co-stained with DAPI both in peripheral and central regions of the fibers; we also observed Trisk95 aggregates that colocalized with RyR1.

In our *CCDC78*-mutated patient, we observed muscular RyR1 aggregates colocalizing with CCDC78. Interestingly, in *CCDC78*-mutated muscle, RyR1 aggregates co-stained with DAPI in both the peripheral and central regions of the fibers, suggesting a localization of RyR1 aggregates in the perinuclear reticulum region (Figure 8e,f). We also observed Trisk95 aggregates colocalizing with RyR1 (Figure 8e,f). The morphometric analysis of the muscle fiber cross-sectional area revealed a significant increase in the percentage of positive nuclei for RyR1 (16 ± 3% vs. 1 ± 0.5% using monoclonal anti-RyR1 antibodies; 15.5 ± 2% vs. 2 ± 1% using polyclonal antibody) and Trisk95 (33 ± 3% vs. 3 ± 1%) in our *CCDC78*-mutated patient compared to the controls (Figure 8d). However, our CCDC78 Co-Ip assay did not reveal any band corresponding to the expected molecular weight for RyR1. Moreover, our confirmation WB study using anti-RyR1 antibodies after CCDC78-Ip did not reveal any interaction between these proteins (Figure 8c).

When analyzing muscular CCDC78 expression by WB in *RYR1*-mutated patients (n = 4) compared to healthy controls (n = 3), we found a significant reduction in the 37 kDa, 48 kDa, and 52 kDa isoforms compared to the controls (70.45% (SD ± 20.15%, *p* < 0.01), 62.93% (SD ± 29.03%, *p* < 0.01), and 57.05% (SD ± 24.82%, *p* < 0.05), respectively). Interestingly, the two *RYR1*-mutated patients harboring a mutation in the CS of the protein (c.130C>T (p.Arg44Cys) and c.1654C>T (p.Arg552Trp)) showed a significant reduction in the 37, 48, and 52 kDa isoforms (73.95% (SD ± 17.93%, *p* < 0.01), 54.34% (SD ± 20.2%, *p* < 0.01), and 49.05% (SD ± 12.99%, *p* < 0.01), respectively) while the two *RYR1*-mutated patients carrying a mutation in the channel and activation core (CAC) (c.11810C>T (p.Ser3937Leu) and c.11708G>A (p.Arg3903Gln)) showed a significant reduction in only the 37- and 52-kDa isoforms (65.94% (SD ± 23.32%, *p* < 0.01), 67.33% (SD ± 31.98%, *p* < 0.01), respectively) (Figure 8a,b). Analysis of muscular RyR1 expression by WB in the *CCDC78*-mutated patient did not reveal significant differences compared to controls.

## 4. Discussion

Our data, obtained by analyzing a family harboring the first *CCDC78* nonsense mutation, shed light on the function of CCDC78 by defining its major interacting proteins and allow us to expand on the muscular phenotype previously associated with the gene. Therefore, we definitively associate this candidate gene with the autosomal dominant CNM4 (OMIM#614807).

We have demonstrated, for the first time, an interaction between CCDC78 and two crucial SR proteins: SERCA1 and CASQ1. SERCA1 actively pumps Ca^2+^ from the cytoplasm to the SR lumen following muscle contraction [31]. At triads, several proteins participate in the ECC process, including CASQ1, a luminal Ca^2+^ binding protein that interacts with RyR1, triadin, and junctin [31]. CASQ1 represents the main Ca^2+^ buffer in the terminal cisternae [32]. Recently, CASQ1 overexpression was found to inhibit Store Operated Calcium Entry (SOCE) by reducing STIM1 clustering and STIM1/OraiI interaction.

The discovered interaction between CCDC78, SERCA1, and CASQ1 strongly supports the role of CCDC78 in the SR and therefore in ECC. Interestingly, we showed that the *CCDC78* mutation led to a loss of or reduction in the interaction between these proteins, suggesting a potential interaction site affected by the mutation.

The reduction in CCDC78 muscular expression by WB in our *RYR1*-mutated patients, especially when the mutations were located in the CS domain, may further support the interaction between CCDC78 and CASQ1, since CS represents the interacting domain of RyR1 with triadin, junctin, and CASQ1 [33,34]. The interaction of CCDC78 with CASQ1 and SERCA1 allows us to conclude that CCDC78 localizes into the SR and supports a possible role of CCDC78 in connecting these two pivotal SR proteins, thus stabilizing the SR and contributing to ECC processes. Notably, the observed SR dilatations in *CCDC78*-mutated muscle corroborate the role of the protein in this cell compartment. Since the protein has been demonstrated to interact with multiple sarcoplasmic proteins, we suggest renaming it *sarcoplasmin* and *SRPM* the related gene.

The interaction with CASQ1 and SERCA1, both proteins largely predominant in fast-twitch (type II) fibers [35,36], together with the results from NADPH diaphorase staining, lead us to conclude that CCDC78 is mainly localized in type II fibers. Interestingly, our mutated patient showed a predominance of type II fibers on muscle biopsy, in line with the observed higher CCDC78 expression in mutated muscle tissue compared to the controls, probably due to a compensatory effect.

In line with our findings, the transcriptome analysis revealed a distinct pattern of DEGs. Indeed, among the most upregulated genes, we found a series of genes involved in the SR and ECC: *TMOD1*, *JPH1*, *CACNA2D1*, *MTM1*, *ASPH*, *CASQ1*, and *ATP2A1*. The upregulation of *CASQ1* and *ATP2A1*, encoding SERCA1, is highly suggestive of a compensatory effect in response to the *CCDC78* mutation, further supporting the intimate role of such CCDC78 interactors. Junctophilin 1, encoded by *JPH1* and located at the triad, mediates the apposition between the ER/SR and plasma membrane in striated muscles [37,38]. *MTM1* encodes for myotubularin, whose balanced expression levels are required for the proper assembly of the SR and TT into triads [31]. The *ASPH* gene encodes three proteins, aspartate beta-hydroxylase, junctin, and junctate: junctin can bind CASQ1 and RyR1 [31]; junctate is a Ca^2+^-sensing structural component of the endoplasmic reticulum (ER)–plasma membrane (PM) junctions, where Orai1 and STIM1 cluster and interact to mediate SOCE [39].

Even among the top downregulated DEGs, we found some genes involved in the SR: *CRACR2A* and *TRDN-AS1*. CRACR2A has a role in the enhancement of Orai1-mediated SOCE [40], thus showing an opposite function compared to CASQ1, in line with the *CASQ1* upregulation observed in our *CCDC78*-mutated muscle. *TRDN-AS1* (Triadin Antisense RNA 1) overlaps with skeletal muscle *TRDN* and is expressed in an opposite orientation to regulate the balance between the cardiac and skeletal muscle isoforms [41]. In the absence of *TRDN-AS1* transcription, TRDN is indeed fully transcribed, giving rise to the skeletal muscle isoform of TRDN (Trisk95 and Trisk51) [42]. Triadin acts as a functional regulator of EEC by interacting with RyRs, junction, CASQ1, and the histidine-rich Ca^2+^-binding protein [31]. Thus, the downregulation of *TRDN-AS1* observed in our *CCDC78*-mutated muscle is in line with our immunofluorescence analysis, showing a significant increase in Trisk95-positive nuclei in mutated muscle fibers.

The involvement of CCDC78 in the triad was previously hypothesized by Majczenko et al. [14]. Our immunostaining study confirms the previous findings of CCDC78 co-localization with RyR1 and desmin in muscular aggregates. Surprisingly, we did not detect an interaction between RyR1 and CCDC78; this was in line with our transcriptome findings, showing no differences in RyR1 expression between *CCDC78*-mutated and control muscles. However, the presence of RyR1 aggregates in *CCDC78*-mutated muscle, the significant reduction in different CCDC78 isoforms, and the CCDC78 aggregates colocalizing with cores in *RYR1*-mutated patients, together with the well-known interaction of RyR1 with CASQ1 [43], likely suggest a possibly indirect interaction between RyR1 and CCDC78. However, further studies are needed to explore this hypothesis.

In addition to the above-described interactions with certain SR proteins, we also discovered that CCDC78 interacts with the following extra-SR proteins: alpha-actin (ACTA1), alpha-actinin-2 (ACTN2), and myosin-1 (MYH1). ACTA1 is the predominant actin isoform in the sarcomeric thin filaments of skeletal muscle [44]. The interaction between CCDC78, involved in the SR, and ACTA1, whose mutations are sometimes associated with an RYR1-like muscular phenotype [45], may suggest a possible role of CCDC78 in connecting ACTA1 to SR structures. This hypothesis is reinforced by the interaction of CCDC78 with ACTN2, an actin-binding protein that crosslinks actin throughout the Z-disk [46,47]. MYH1, a cytoskeletal muscular protein interacting with actin [48], has not been clearly associated with RYR1-related myopathies. However, *MYH1* mutations have been linked to non-exertional rhabdomyolysis in horses [49], and *MYH1* was recently considered a candidate gene for recurrent rhabdomyolysis in humans [50]. Interestingly, Eckhardt et al. [51] showed a quantitative reduction in MYH1 in a mouse model with biallelic *RYR1* mutations; accordingly, Wang et al. [52] showed a significant reduction in MYH1 expression in muscle tissue from *RYR1*-mutated patients and suggested MYH1 as a potential biomarker for RYR1 myopathies. These data, together with the found interaction with CCDC78, reinforce the hypothesis of an intimate role of MYH1 with other SR proteins.

A muscular clinical phenotype associated with *CCDC78* mutations was previously described by Majczenko et al. [14]; the phenotype included early-onset distal muscle weakness, myalgia, easy fatigue, mild cognitive impairment, and normal CPK levels. Differently from Majczenko et al., our *CCDC78*-mutated patient showed adult-onset myalgias and painful muscle cramps with bilateral calf hypertrophy and persistent moderate CPK elevation; the patient’s daughter, harboring the same mutation, was asymptomatic at 19 years of age and showed only bilateral calf hypertrophy. No cognitive impairment was detected in our patients. Moreover, our patient did not show any core-like lesions, differently from the previous study; more interestingly, ultrastructural analysis revealed SR abnormalities previously described only in ccdc78 zebrafish morphants harboring a splice-site mutation [14]. These data may suggest a more prominent muscle involvement associated with the nonsense variants, thus suggesting a likely haploinsufficiency effect.

However, we cannot exclude that such a slightly different phenotype could be mediated by the different variant effect on different *CCDC78* isoforms. Indeed, in different control tissues, including muscle, we surprisingly found two alternative in-frame transcripts. As previously demonstrated [53], individual mammalian genes often produce multiple mRNA and protein isoforms that may have related, distinct, or even opposing functions. It is possible that CCDC78 products with different lengths may act in different roles in muscle and other tissues; consequently, different mutations could lead to a kaleidoscopic effect on phenotypes. This hypothesis is in line with the findings of a series of CCDC78 interactors inside and outside the SR, which highly indicates a role in different cellular compartments.

To further complicate the understanding, we have provided evidence that the nonsense *CCDC78* variant results in a mutant allele with a premature stop codon, which is likely mostly degraded on the mRNA level through NMD. However, we observed 84% of the 48 kDa isoform expression in the mutated patient compared to the controls, higher than the expected 50%, possibly indicating some degree of expression compensation through increased wild-type translation. Moreover, our WB study revealed a significant reduction in the 52 kDa isoform in the mutated patient. Since the mutation results in a missense variant (c.1210G>A, p.Glu404Lys) in the NM_001378030.1 transcript, this reduction likely reflects a dominant negative effect driven by the missense variant. Missense mutations can indeed affect DNA transcription factors, resulting in altering the expression of the corresponding protein. In addition to reduced transcription or unstable mRNA affecting protein expression, disease-causing missense mutations are believed to cause intracellular retention of their respective mutant proteins [54,55].

In addition to the significant processes supporting the prominent role of CCDC78 in the ECC, such as “muscle contraction”, “calcium ion transport into cytosol”, “response to muscle activity”, and “endoplasmic reticulum organization”, we also found, through the GO analysis, a series of significant processes related to cell division, cytoskeleton organization, and myogenesis. These include “positive regulation of cell migration”, “negative regulation of cell proliferation”, “muscle organ development”, “actin filament organization”, “cytoskeleton organization”, and “negative regulation of skeletal muscle cell differentiation”. Klos Dehring et al. [56] demonstrated in a multi-ciliated cell model that CCDC78 represents a centriole-associated and deuterosome protein that is essential for the amplification of centrioles; they are microtubule-based structures involved in centrosomes, cilia, and flagella formation. Some recent studies indicate a possible role of cilia in muscle progenitors [57]. Recently, Palla et al. [58] found that the ability of muscle stem cells to regenerate is regulated by the primary cilium. Our RNA-seq results suggest a possible role of CCDC78 in cell division processes. Furthermore, the colocalization of CCDC78 and gamma-tubulin in HeLa cells during cell division highly suggests a possible role of the protein in muscle cell division. Moreover, among the top downregulated DEGs, we identified the following genes involved in cytoskeleton organization: *AHNAK2*, *CDH23*, and *KIF7*. AHNAK2 is a costameric protein showing co-localization with α-actinin [59]; CDH23 is a member of the cadherin superfamily, implicated in stereocilia organization and hair bundle formation [60]; and *KIF7* encodes a cilia-associated protein within the kinesin family [61].

## 5. Conclusions

In conclusion, our findings shed light, for the first time, on interactors and possible role of CCDC78 in skeletal muscle, definitely locating the protein in the SR. Moreover, our data broaden the phenotype previously associated with *CCDC78* mutations, revealing possible histopathologic hallmarks of disease in humans. Future studies including an increased number of patients harboring various *CCDC78* mutations are needed to better define the exact associated phenotype and the precise role of CCDC78 in skeletal muscle, within both the SR and other subcellular structures.

## Data Availability

The data that support the findings of this study are available from the corresponding author upon reasonable request.

## Supplementary Materials

The following supporting information can be downloaded at: https://www.mdpi.com/article/10.3390/cells13171504/s1, Figure S1: Comparison of gene expression between the defined groups of samples; Figure S2: Sanger analysis of the *CCDC78* variant in the patient (a), patient’s daughter (b), and patient’s mother (c). Table S1: List of the analyses performed in patients and control subjects (wild type); Table S2: List of primers used to amplify exons 10–14 region of *CCDC78* gene; Table S3: Identified possible CCDC78 partners by nLC-nESI-HRMS/MS analysis.
