# Supplementary material for: CCDC78: Unveiling the Function of a Novel Gene Associated with Hereditary Myopathy
Source: Cells. 2024 Sep 8;13(17):1504. doi: 10.3390/cells13171504 (PMC11394131; doi:10.3390/cells13171504)
Supplement: Supplementary file 1 [file cells-13-01504-s001.zip › Supplementary Information_7.9.24.pdf]

Supplementary Materials

Materials and methods

Table S1. List of the analyses performed in patients and control subjects (wild type).

Subject	Sex	Age	Muscle biopsy site	genetic variant	WB study	RT-qPCR (muscle tissue)	RT-qPCR (lymphocytes)	RNA-seq	IF	Co-Ip
patient	M	50	left rectus femoris	*CCDC78*: c.1206G>A (p.Trp402*)	✓	✓	✓	✓	✓	✓
WT1	M	55	left rectus femoris	-	✓				✓	✓
WT2	M	47	right rectus femoris	-	✓				✓	✓
WT3	M	45	left rectus femoris	-	✓				✓	✓
WT4	M	48	left rectus femoris	-	✓	✓	✓	✓		
WT5	M	62	left rectus femoris	-	✓	✓	✓	✓		
WT6	M	54	left rectus femoris	-	✓	✓	✓	✓		
R1	F	40	right rectus femoris	*RYR1*: c.11810C>T (p.Ser3937Leu)	✓				✓	
R2	M	37	left rectus femoris	*RYR1*: c.130C>T (p.Arg44Cys)	✓				✓	
R3	M	47	right rectus femoris	*RYR1*: c.1654C>T (p.Arg552Trp)	✓				✓	
R4	M	60	left rectus femoris	*RYR1*: c.11708G>A (p.Arg3903Gln)	✓				✓	

Legend: WB, Western Blotting study; Co-Ip, Coimmunoprecipitation study; IF, immunofluorescence analysis, RNA-seq, Transcriptome profiling; WT, wild type; R, *RYR1*-mutated patient.

Morphological analysis of muscle biopsies and HeLa cells

For ultrastructural studies, small samples were fixed in 2.5% glutaraldehyde, pH 7.4, postfixed in 2% osmium tetroxide for 2 hours, dehydrated and embedded in epoxy resin. At least 3 blocks from each sample were studied, including longitudinal and transverse-oriented samples. Semi-thin sections were stained by toluidine blue and examined with a Zeiss Axiomat light microscope to select pathological areas. Ultrathin sections were stained with uranyl acetate and lead citrate. The grids were observed using a Zeiss EM 109 electron microscope (80 kV; Karl Zeiss, Berlin, Germany).

DNA analysis

WES was performed by the NovaSeq6000 (Illumina) on DNA from peripheral blood samples. Mutational analysis was carried on using GATK version v.4.0. External datasets, such as 1000 genomes, ExAC and GnomAD, were used to define novel variants, not previously identified. We performed a prioritization of the variants selecting frameshift, splice, stopgain or stoploss mutations, missense variants predicted to be damaging by CADD-phred prediction tools and variants with minor allele frequency (MAF) < 0.01. Single nucleotide variants were confirmed by Sanger sequencing. The American College of Medical Genetics and Genomics (ACMG) criteria were used to classify detected variants. Putatively deleterious variants were validated by PCR-based standard capillary Sanger sequencing.

The exons and adjacent intron regions of *CCDC78* gene were amplified by PCR using primers specific for *CCDC78* gene. The PCR were sequenced in both forward and reverse directions by automated sequencer ABI 3500 (Applied Biosystems). The results were analyzed using Chromas version 2.33 software and compared with reference sequence NG_032932.1. Mutation was numbered according to the published cDNA sequence NM_001378030.1 for the longer transcript and according to NM_001031737.3 for the shorter transcript.

Primary fibroblast cultures

Primary cultures of fibroblasts, obtained from healthy control skin biopsies were grown in standard condition with 1 ml of Dulbecco's modified Eagle's medium (Merck KGaA, Darmstadt, Germany) supplemented with 10% fetal calf serum (Merck KGaA), 1% L-glutamine, and 1% streptomycin-penicillin (100 IU/ml and 100 μg/ml, respectively; Merck KGaA). Flasks were maintained at 37°C in a humidified atmosphere containing 5% CO_2. Analysis of subconfluent fibroblasts cultures was carried out after 15 replications. At this time, cells were treated with 2 cc of trypsin and harvested in complete medium.

HeLa cells

HeLa cells were grown in a 5% CO_2 atmosphere in Dulbecco's modified Eagle's medium high glucose (DMEM; Euroclone), supplemented with 10% fetal bovine serum (Gibco), 100 U/ml penicillin and 100 mg/ml streptomycin.

Transcripts analysis
PCR amplifications were performed with primers surrounding the mutated region (Tab S2).

Table S2. List of primers used to amplify exons 10-14 region of *CCDC78* gene

CCDC78-10/11RNAFW3	CAGGCAGTGGAGCACGCAGAT
CCDC78-11/12RNAFW	GGAGGACCAGCACGGCGG
CCDC78-12/13RNAFW	CATCAGAGCCACAGGGCCTG
CCDC78-14RV	CGTGCTTGTACCTGCCCAGGT
CCDC78-14RV2	TCAGGATTTCGTGCTTGT

Transcriptome profiling
Sequence reads were trimmed to remove possible adapter sequences and nucleotides with poor quality using Trimmomatic v.0.36. The trimmed reads were mapped to the Homo sapiens GRCh37 reference genome available on ENSEMBL using the STAR aligner v.2.5.2b, a splice aligner that detects splice junctions and incorporates them to help align the entire read sequences. Unique gene hit counts were calculated by using featureCounts from the Subread package v.1.5.2. The hit counts were summarized and reported using the gene_id feature in the annotation file. Only unique reads that fell within exon regions were counted.

RT-qPCR
Light cycler protocol included 10 min of initial reverse transcription at 50°C followed by 2 min at 95°C. The following step was 40 cycles of 5 s each at at 95°C denaturation and 30 s at 60°C of primer annealing, extension, and relative fluorescence unit data collection. Data were analyzed with CFX Manager Software V3.0 (Bio-Rad, California, USA). *CCDC78* expression (qHsaCED0038732) was compared to the expression of the reference genes. We evaluated the stability of potential reference genes in muscle tissue and PBLs: hypoxanthine phosphoribosyltransferase 1 (*HPRT1*, qHsaCID0016375), serpin peptidase inhibitor, clade C, member 1 (*SERPINC1*, qHsaCID0021147), glucuronidase beta (*GUSB*, qHsaCIP0028142), connective tissue growth factor (*CTGF*, qHsaCED0002044), transferrin receptor (*TFRC*, qHsaCID0022106) and zinc finger protein 80 (*ZNF80*, qHsaCED0018708). *HPRT1*, *SERPINC1* and *ZNF80* were selected for data normalization, as they showed the most constant expression and stability. Relative quantification of gene expression was evaluated using the comparative threshold cycle value method 2–ΔΔCt.

Western blotting study
Total protein concentration was measured using QubitTM protein assay kit (Thermo Scientific, Rockford, USA). We prepared 50 μg protein for each sample through 5%, 12% and 4–15% Mini-PROTEAN® TGX™ precast gels (Bio-Rad, California, USA), and electrophoretically transferred to nitrocellulose membranes (Bio-Rad, California, USA). Membranes were blocked 1 h at room temperature (RT) in TBS containing 5% skimmed milk

and 0.2% Tween 20, incubated overnight at 4°C with primary antibodies in 5% BSA and 0.2% Tween 20, and 1 h at RT with secondary antibodies in TBS containing 5% skimmed milk and 0.1% Tween 20. Antibodies were diluted as follows: 1:1700 rabbit anti-CCDC78 (AV53233, Sigma-Aldrich) 1:3300 rabbit anti-RyR1 (Rossi, Bencini, et al. 2014), 1:20000 mouse anti-ß-actin (Santa Cruz Biotechnology, USA), 1:15000 mouse anti-GAPDH (39-8600, Thermo Scientific, Massachusetts, USA), 1:30000 (primary anti-CCDC78) and 1:80000 (primary anti-RyR1) peroxidase affinipure goat anti-rabbit IgG (111-035-045, Jackson ImmunoResearch Laboratories, Pennsylvania, USA) and 1:50000 peroxidase affinipure goat anti-mouse IgG (115-035-062, Jackson ImmunoResearch Laboratories, Pennsylvania, USA). Proteins were revealed by Clarity MaxTM Western ECL Substrate (Bio-Rad, California, USA) according to the manufacturer's instructions and images acquisition from Gel Doc 2000 Imaging System (Bio-Rad, California, USA) were performed with Quantity One® 1-D analysis software.

Co-immunoprecipitation assay

Fifty mg of muscle tissue from healthy controls and CCDC78 patient were lysed over ice using a Potter-type tissue homogenizer with IP Lysis/Wash Buffer (Thermo Scien-tific, Rockford, IL, USA) and Complete Mini Anti-protease Cocktail Tablets (Roche Applied Science, Laval, PQ, Canada) according to the manufacturer's' instructions.

Lysates were incubated on ice for 5 minutes with periodic mixing and then cell debris were removed by centrifugation at 13,000×g for 10 minutes. Total protein concentra-tion was measured using Qubit protein assay kit (Thermo Scientific). Co-Ip assay was performed using the Pierce Crosslink Magnetic IP/Co-IP Kit (Thermo Scientific, Rock-ford, IL, USA). Anti-CCDC78 (AV53233, Sigma-Aldrich) was diluted to the final concentration of 10µg/100µL. We used the crosslinking chemistry of disuccinimidyl su-berate (DSS) to performe co-IP by coupling antibodies to the beads covalently. The ly-sate solution, diluted to 500µL with IP Lysis/Wash Buffer to a final concentration of 5 ng/ul of protein, was added to the tube containing crosslinked magnetic beads and in-cubated for 2,5 hours at 4°C on a rotator. Then we collected the beads and removed the unbound sample.

The immunoprecipitated protein was loaded in 5%, 12% and 4-15% gels. Gel was fixed (fixing solution: 50% methanol and 10% glacial acetic acid) for 1 hr with gentle agita-tion and then it was stained (staining solution: 0.1% Coomassie Brilliant Blue R-250, 50% methanol and 10% glacial acetic acid) for 20 min with gentle agitation. After-wards the gel was destained (destaining solution: 40% methanol and 10% glacial acetic acid) and stored (storage solution: 5% glacial acetic acid) for mass spectrometry analy-sis.

For each immunoprecipitation assay, positive controls of protein expression at the whole lysate before co-IP and negative controls with nonspecific IgG (111-035-045, Jackson ImmunoResearch Laboratories, Pennsylvania, USA) were performed.

Co-IP elution (25-50 ul) were separated on 5%, 12% and 4-15% Mini-PROTEAN® TGX™ precast gels using methods described above. For protein blotting, the following primary antibodies were used: 1:1700 rabbit anti-CCDC78 (AV53233, Sigma-Aldrich), 1:3300 rabbit anti-RyR1 (Rossi, Vezzani, et al. 2014), 1:15000 mouse anti-GAPDH (39-8600, Thermo Scientific), 1:1000 mouse anti-SERCA1 (VE121G9, Thermo Scien-tific), 1:3000 rabbit anti-ACTN2 (HPA008315, Sigma-Aldrich), 1:500 rabbit anti-TPM1 (HPA000261, Sigma-Aldrich),

1:1000 rabbit anti-CASQ1 (HPA026823, Sigma-Aldrich), 1:500 rabbit anti-TPM2 (11038-1-AP, Thermo Scientific), 1:1000 rabbit anti-MYH1 (ZRB1214, Sigma-Aldrich), 1:1000 mouse anti-ACTA1 (SAB4200602. Sigma-Aldrich), peroxidase affinipure goat anti-rabbit IgG (111-035-045, Jackson ImmunoResearch Laboratories) and peroxidase affinipure goat anti-mouse IgG (115-035-062, Jackson ImmunoResearch Laboratories).
Proteins were revealed by Clarity MaxTM Western ECL Substrate (Bio-Rad) according to the manufacturer's instructions and images acquisition from Gel Doc 2000 Imaging System (Bio-Rad) were performed with Quantity One® 1-D analysis software. For each immunoprecipitation assay, positive controls of protein expression at the whole lysate before co-IP and negative controls with nonspecific IgG (111-035-045, Jackson ImmunoResearch Laboratories, Pennsylvania, USA) were performed. GAPDH was used as a negative control to demonstrate the specificity of Co-Ip.

Mass spectroscopy study
The blue Coomassie stained 1D-gel was used for protein analysis. Seven bands of interest were selected and have been manually excised from the gel using a clean scalpel blade for the following mass spectrometric analyses. Each band of interest was transferred into a 1.5 mL low-binding Eppendorf tube for destaining and successive reduction, alkylation, and enzymatic digestion.
Destaining was performed by adding 120 µL of ACN to the gel band, sufficient to cover the gel piece, and left for ten minutes; then the ACN was discarded and 100 µL 0.1 M NH4HCO3 were added. After 5 minutes NH4HCO3 solution was removed. These two steps were repeated two times or more until the supernatant was no more colored. A final 100 µL volume of ACN was added and then discarded and the residual ACN was evaporated into a centrifugal evaporator concentrator (Jouan, Thermo Fisher). To the gel band, 100 µL of 10 mM DTT in 0.1 M NH4HCO3 were added and the Eppendorf tube was left at 56 °C for 45 min in an orbital shaker. The sample was taken to room temperature and the solution removed; 130 µL of ACN were added, vortexed, and then immediately discarded. Alkylation was done by adding 100 µL of 55 mM iodoacetamide in 0.1 M NH4HCO3; the solution was shaken for 30 min at room temperature, protected from the light. The iodoacetamide solution was removed and then 100 µL of ACN were added, then discarded; 100 µL of 0.1 M NH4HCO3 were added and, after shaking for 10 minutes, discarded. These steps were repeated two times and then 100 µL of 100% ACN were added to each tube. After shaking for 5 min, the ACN was removed and left to evaporate the residual ACN for 20 min. The enzymatic digestion was performed by adding 50 µL of 50 mM NH4HCO3 and 1 µL of 0.4 µg/µL LysC solution in ultrapure water. The sample was left for 3 h at 37 °C under shacking. Then 1 µL of 0.5 µg/µL trypsin solution in 50 mM acetic acid was added and left to incubate overnight at 37 °C under shacking. The reaction was stopped by adding 1 µL of 10% TFA and the pH was measured to ensure that it was around 3; more 10% TFA was added if necessary. The sample was centrifuged and the supernatant was used for C18 STAGE tip purification, following the procedure described by Rappsilber et al.
The C18 STAGE tip was eluted directly in a polypropylene conical insert with 50 µL of 0.5% acetic acid in H2O:ACN 20:80 (v:v). The solution was concentrated under vacuum in a centrifugal evaporator to approximately 10 µL and then the final volume was brought to 20

µL with 0.5% acetic acid. The final solution of the digested proteins was used for mass spectrometry experiments.

nLC-nESI-HRMS/MS

Excalibur softwares were used for instruments control and data acquisition (version 4.2 for EASY-nLC and version 2.0.7 for LTQ Orbitrap).The nESI interface parameters were: nESI spray potential 1.7 kV, capillary and tube lens voltages were 42 and 120 V, respectively. MS data acquisition was done in DDA (data dependent acquisition) mode, performing a HRMS (60000 nominal resolution, at m/z 400) full scan from 350 to 2000 m/z in the Orbitrap analyzer, using a 1 x 106 target value. MS/MS spectra were recorded in the linear quadrupole ion trap analyzer: precursor ions were selected from the 7 most intense signals in the HRMS full scan spectrum above 600 a.u. threshold and with an isolation window of 2.2 Da. Normalized collision energy of 35% and 20 ms activation time were used. Singly charged or no charge state assigned precursor ions did not trigger MS/MS experiments. Dynamic exclusion of already selected precursor masses was applied, with an exclusion time of 30 s and an exclusion window of 20 ppm (repeat count 2, repeat duration 15 s). The chromatographic column was an AcclaimÒ PepMap 100 C18, 3 µm, 100 Å, 75 µm x 150 mm (Dionex Thermo), operating at 0.3 µL/min flow rate. Solvent A was 100 % water and solvent B was 80% acetonitrile/20% water, both containing 0.1% formic acid; solvents were of LC-MS grade from Sigma (Sigma Italy, Merck). A 1 µL volume was injected into the nLC-nESI HRMS/MS system. Elution was done by gradient starting from 2% B for 5 min, to 40% B in 340 min, to 90% B in 5 min and then returned to initial conditions.

Results

RNA-seq analyses

Although statistically significant only according to p-values and not adjusted p-values, among the subsequent 647 processes we found: positive regulation of skeletal muscle fiber development (488th position), positive regulation of muscle cell differentiation (517th), muscle cell development (541th), striated muscle cell differentiation (581th), skeletal muscle fiber development (638th), intrinsic apoptotic signaling pathway in response to endoplasmic reticulum stress (646th), regulation of muscle contraction (651th), negative regulation of muscle hypertrophy (833th), positive regulation of fast-twitch skeletal muscle fiber contraction (850th), establishment of protein localization to endoplasmic reticulum (926th), negative regulation of muscle tissue development (942th), negative regulation of skeletal muscle hypertrophy (953th), endoplasmic reticulum calcium ion homeostasis (984th), muscle filament sliding (998th), skeletal muscle tissue regeneration (1013th).

Mass spectroscopy study

Table S3. Identified possible CCDC78 partners by nLC-nESI-HRMS/MS analysis.

Band n.	Accession	Score	Mass	Num. of significant matches	Num. of significant sequences	emPAI	Description
1	MYH1_HUMAN	4021	223006	200	86	3,27	Myosin-1 GN=MYH1
2	AT2A1_HUMAN	286	110182	19	13	0,46	Sarcoplasmic/ endoplasmic reticulum calcium ATPase 1 GN=ATP2A1
3	ACTN2_HUMAN	2086	103788	98	44	4,03	Alpha-actinin-2 GN=ACTN2
4	CASQ1_HUMAN	168	45132	8	5	0,42	Calsequestrin-1 GN=CASQ1
5	ACTS_HUMAN	2427	42024	133	22	9,43	Actin, alpha skeletal muscle GN=ACTA1
6	TPM2_HUMAN	1211	32831	65	23	10,1	Tropomyosin beta chain GN=TPM2
7	TPM1_HUMAN	1327	32689	78	29	21,22	Tropomyosin alpha-1 chain GN=TPM1

Legend: emPAI (exponentially modified protein abundance index); Score (overall Protein Score reflects the combined scores of all observed mass spectra that can be matched to amino acid sequences within that protein. A higher score indicates a more confident match).

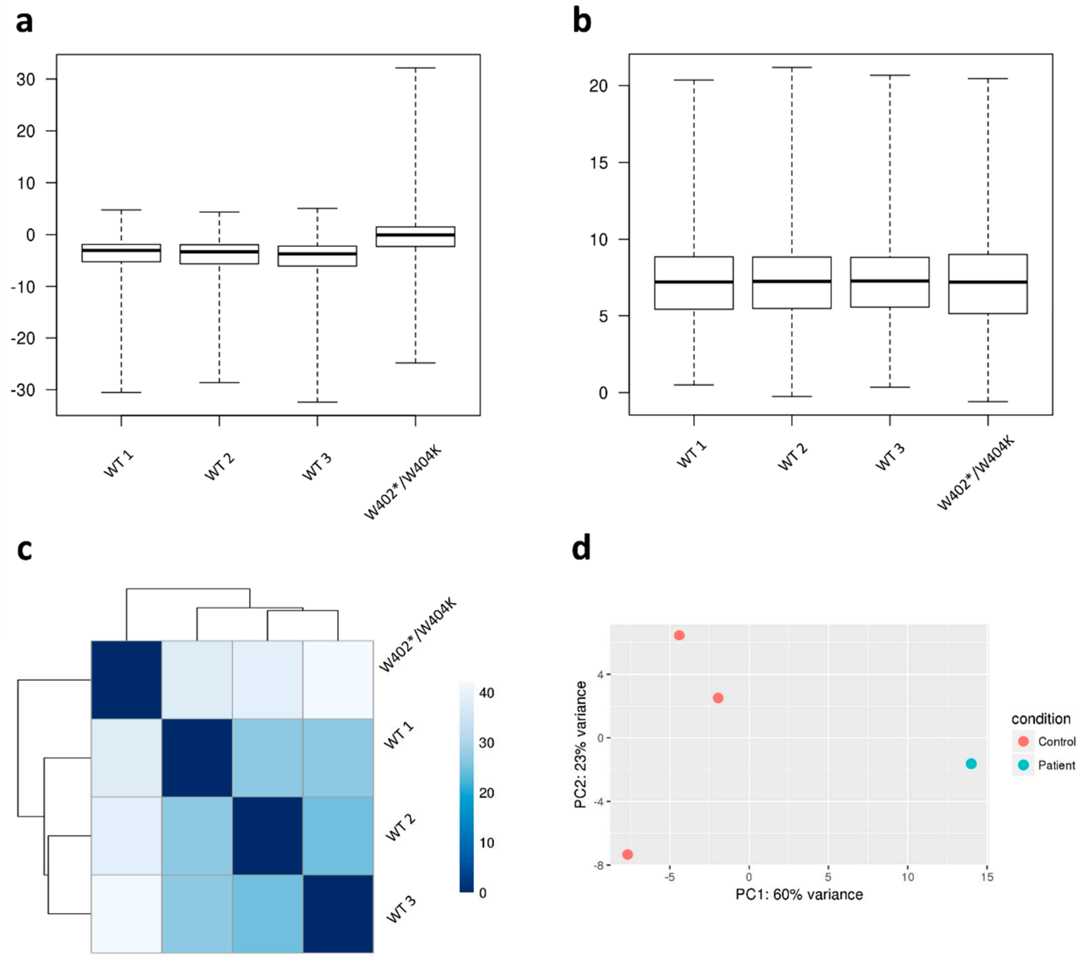

Figure S1. Comparison of gene expression between the defined groups of samples. The box plot analysis provides a visual representation of raw **(a)** and normalized **(b)** expression values. RNA-sequencing sample distance analysis **(c)**: RNA-sequencing count tables were statistically analyzed with DESeq2 (Love, Huber, and Anders 2014), and the Euclidean distances were calculated between each sample; samples were clustered using hierarchical clustering analysis, and the dendrograms represent the clustering results. The heatmap illustrates the pairwise distances between the indicated samples, with the colors indicating the distances as shown in the key in the right; i.e., the more blue the square, the more similar the samples). **(d)** Principal component analysis: PC1 and PC2 variance of expression between RNA-seq samples is shown. The plot shows the projection of the samples onto the two-dimensional space spanned by the first and second principal components of the covariance matrix. The expression levels used as input are normalized log CPM values.

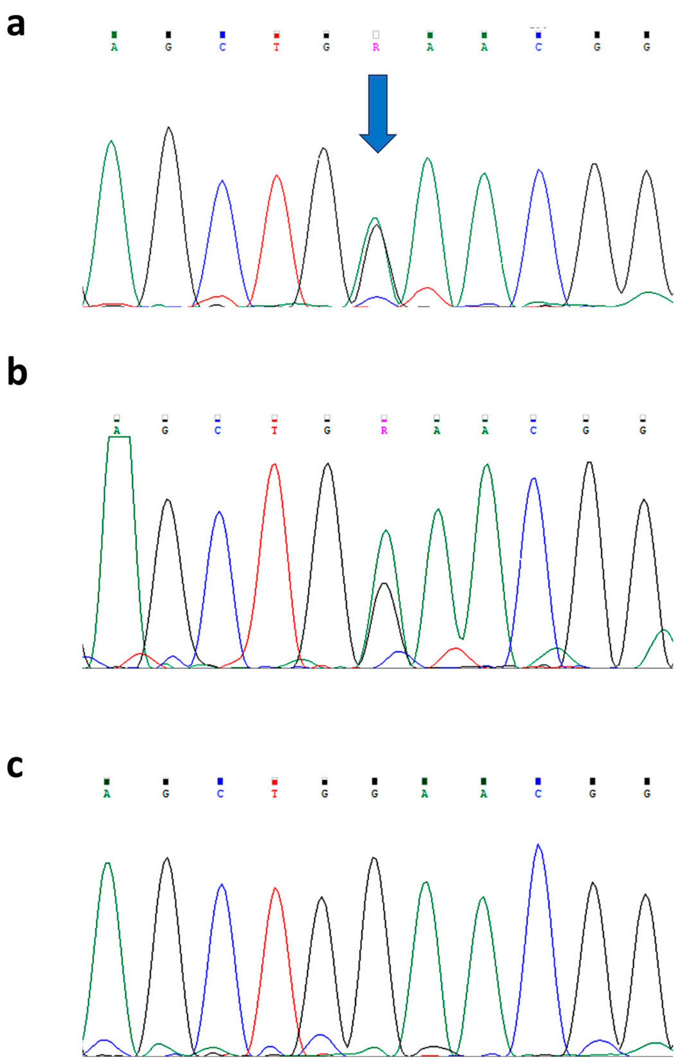

Figure S2. Sanger analysis of the *CCDC78* variant in the patient (a), patient’s daughter (b), patient's mother (c).
